# Pennsylvania

**Published:** 1896-09

**Authors:** 


*Pennsylvania.* State Veterinarian Pearson finds much of his time occupied in doing missionary work among farming districts remote from cities, where the use and value of tuberculin are little known and the dangers of tuberculous cattle little appreciated. He should have the active aid of every individual veterinarian in this work.

A Farmers' Protective Association at Newtown, near Columbia, gives forth the statement that, inasmuch as the danger of transmission of the disease to the human family by means of milk has not been sustained, the municipal legislation against them is too severe in view of the fact that the State does not acknowledge the disease to be worthy of prohibitive legislation. A strong point here for future legislators, and how necessary missionary work still remains as a necessity.

Eleven out of sixteen high-priced cattle is the record of one herd in Montgomery County.

Three out of a herd of seventeen responded in Centre County.

Cumberland County finds tuberculosis among her cattle. New Brighton in Western Pennsylvania; Alverton, Westmoreland County.

The Philadelphia *Times* well says in its issue of August 5th: "The public health must be maintained even at the pecuniary loss of individual citizens, and the farmer should be just in his criticism of the State Veterinarian in the performance of his duty."

The Philadelphia *Press* in its issue of August 10th, referring to the formation of a Farmers' Protective Association to oppose the tuberculin-test, says such work is directly in the interests of the artificial dairies near the cities, where milk in greater quantities may be produced cheaper by stimulating foods cheaply obtained.

Huntingdon and Blair Counties are added to the list of those seeking inspections of cattle through the Live-stock Sanitary Board.

A public pasture lot at New Brighton, in the western part of the State, where some twenty-three cattle were pastured belonging to as many owners, has developed as another disease-centre.

Cerebro-spinal meningitis in enzoötic form is reported from a section of Chester County.

